# Scent-sniffing dogs can discriminate between native Eurasian and invasive North American beavers

**DOI:** 10.1038/s41598-019-52385-1

**Published:** 2019-11-04

**Authors:** Frank Rosell, Hannah B. Cross, Christin B. Johnsen, Janne Sundell, Andreas Zedrosser

**Affiliations:** 1 Faculty of Technology, Natural Sciences, and Maritime Sciences, Department of Natural Sciences and Environmental Health, University of South-Eastern Norway, Bø in Telemark, Norway; 2 Lammi Biological Station, University of Helsinki, Pääjärventie 320, 16900 Lammi, Finland

**Keywords:** Conservation biology, Coevolution

## Abstract

The invasion of a species can cause population reduction or extinction of a similar native species due to replacement competition. There is a potential risk that the native Eurasian beaver (*Castor fiber*) may eventually be competitively excluded by the invasive North American beaver (*C. canadensis*) from areas where they overlap in Eurasia. Yet currently available methods of census and population estimates are costly and time-consuming. In a laboratory environment, we investigated the potential of using dogs (*Canis lupus familiaris*) as a conservation tool to determine whether the Eurasian or the North American beaver is present in a specific beaver colony. We hypothesized that dogs can discriminate between the two beaver species, via the odorant signal of castoreum from males and females, in two floor platform experiments. We show that dogs detect scent differences between the two species, both from dead beaver samples and from scent marks collected in the field. Our results suggest that dogs can be used as an “animal biosensor” to discriminate olfactory signals of beaver species, however more tests are needed. Next step should be to test if dogs discern between beaver species in the field under a range of weather conditions and habitat types and use beaver samples collected from areas where the two species share the same habitat. So far, our results show that dogs can be used as a promising tool in the future to promote conservation of the native beaver species and eradication of the invasive one. We therefore conclude that dogs may be an efficient non-invasive tool to help conservationist to manage invasive species in Europe, and advocate for European wildlife agencies to invest in this new tool.

## Introduction

The invasion of a species into an ecosystem can cause population reduction or extinction of a related native species due to replacement competition^1,2^. Species that occupy a similar ecological niche will compete with one another for resources that are limited in supply. Alien mammals directly threaten a significant number of native species in Europe^3^. For example, the invasive North American grey squirrel (*Sciurus carolinensis*) has gradually replaced populations of native red squirrels (*S. vulgaris*) in Great Britain, Ireland, and Italy^4,5^. This replacement has been caused by direct competition for food resources between the two species, which has reduced juvenile body growth, recruitment, and female breeding success in the red squirrel^4,6^. In addition, grey squirrels are also host to a parapox virus that causes a fatal disease in red squirrels^7,8^. Other examples of competitive interactions between native and introduced species are the European mink (*Mustela lutreola*) and the introduced American mink (*Neovision vision*)^9^ in Europe, or the endangered marsupial carnivore spotted-tailed quoll (*Dasyurus maculatus*) and introduced eutherian carnivores, such as the red foxes (*Vulpes vulpes*), wild dogs (*Canis lupus* ssp.) and feral cats (*Felis catus*) in Australia^10^.

Two species of beaver (*Castor* spp.) exist in Eurasia today, the native Eurasian beaver (*C. fiber*, from now on referred to as *Cf*) and the invasive North American beaver (*C. canadensis*, from now on referred to as *Cc*). *Cc* was introduced to Europe in 1937 to supplement an ongoing reintroduction of *Cf* to Finland from Norway^11^. At this time, it was assumed that *Cc* and *Cf* were the same species. However, in 1973 it was determined, based on different chromosome numbers, that the genus *Castor* consisted of two species (*Cf* = 48 chromosomes, *Cc* = 40)^12^. Today, we find *Cc* in several regions in Russia and Finland, and small populations exist at various locations in Germany, Hungary, Luxembourg and Belgium due to illegal releases or escapes from zoological collections^13,14^. In these areas, the two species occur in close proximity and to some extent overlap^14^. The species are very similar in morphology, behavior and ecology^14,15^, which suggests that they should compete when sympatric. There is evidence that *Cf* is the weaker competitor, and *Cc* may colonize vacant habitats faster due to its greater fecundity^14^. This suggests that there is a potential risk that *Cc* may eventually competitively exclude *Cf* completely. An invasion of *Cc* would be detrimental to any country in Europe as most national conservation laws and international treaties forbid the spread of alien species. It has been advocated that the precautionary principle be adhered to, and an eradication strategy of *Cc* from Eurasia has been outlined^14^.

Both beaver species are highly territorial, semi-aquatic rodents that live in family units (commonly referred to as colonies)^16,17^. They defend their territories by scent marking with castoreum from the castor sacs^18,19^. Via behavioural bioassays Peterson, *et al*.^20^ found that Cc was unable to recognize another beaver species by castoreum, as response frequency did not differ significantly to secretions from conspecifics or heterospecifics. However, a lack of discrimination does not necessarily mean there is no difference^20^. It is therefore unknown whether the chemical signal of castoreum also differs between the two species.

Several methods can be used to determine which species of beaver is present at a location without harvesting them. The two most reliable methods are to examine the color and viscosity of anal gland secretion in live-trapped and correctly sexed beavers^21^, and/or DNA analysis of hairs collected in hair traps^22,23^. Both methods are costly and time consuming.

Dogs (*Canis lupus familiaris*) are a possible non-invasive alternative for species discrimination since they have an extensive sense of smell^24–26^, and are being used extensively as field assistants by researchers, conservationists and managers. Dogs are used to locate live or dead animals, their dens, nests or lairs, and their signs and scats^27–29^. They can detect and discriminate between scats from different fox species (*Vulpes macrotis, V. vulpes* and *V. cinereoargentatus*)^30,31^ and bear species (*Ursus arctos* and *U. americanus*)^32^, and badger (*Meles meles*) scats from raccoon dog (*Nyctereutes procyonoides*) scats^33^. Dogs have also shown great potential in locating invasive species like Australian brushtail possums (*Trichosurus vulpecula*) in New Zealand^34^, brown treesnakes (*Boiga irregularis*) in Guam^35^, and Burmese python (*Pyton bivittatus*) in the USA^27^.

In this study, we investigated the potential of dogs as a conservation tool to determine, by sniffing out beaver castoreum scent samples, whether *Cf* or *Cc* are present at a specific beaver colony. We carried out two discrimination experiments, one with castoreum samples from dead beavers (experiment one) and one with castoreum scent marks collected in the field (experiment two). We hypothesized that dogs can discriminate between the castoreum of the two beaver species. For experiment one, we predicted that the dogs would indicate on the beaver species they are imprinted on and ignore the control samples. For experiment two, we predicted that the dogs would indicate on the beaver species they are imprinted on, or if the imprinted species was not present, that the dogs would return to their handler and sit.

## Results

### Experiment one: castoreum samples from dead beavers

All seven dogs carried out 10 trials each and successfully discriminated between the two beaver species (i.e. indicated only on target beaver scent) via castoreum from dead beavers with an average accuracy of 96.7% (SD ± 3.3), sensitivity of 90% (SD ± 10.0) and a specificity of 98% (SD ± 2.0).

### Experiment two: castoreum scent marks from beavers

The four dogs with further training carried out 27 testing trials each and successfully discriminated between the castoreum scent marks from both beaver species (i.e. indicated only on target beaver scent) with an average accuracy of 97.8 (SD ± 1.9), sensitivity of 83.3% (SD ± 14.3) and a specificity of 99.7% (SD ± 0.7). When presented to the castoreum of the non-target species, all dogs but one (which rejected the non-target in eight out of nine trials) correctly rejected (i.e. returned to the handler) the scent in nine out of nine trials, and during blank trials none of the dogs indicated on blanks.

## Discussion

Our hypothesis that dogs can discriminate castoreum from the two beaver species (i.e. the Eurasian and the North American beaver), was supported. We showed that dogs detected scent differences between the two beaver species, both from castoreum from dead male and female beavers and from scent marks collected in the field. Our results suggest that dogs can be used as an “animal biosensor” to discriminate between the two beaver species scent samples, i.e. scent collected from dead animals and their scent marks. Also, it suggests that they can recognize and indicate on volatile compounds of different ages.

To our knowledge, few studies have evaluated the discrimination rate of individual dogs trained to find scents of species belonging to the same genus. In controlled discrimination experiments, Smith, *et al*.^36^ showed that four dogs were 100% accurate at discriminating kit fox scats from red foxes scats. However, the dogs were less accurate (67%) to ignore red fox scats when only these scats were present in a trial. In our study, the dogs were more accurate at ignoring the scent marks of the non-target species (>96%). Hurt, *et al*.^32^ showed that three dogs successfully (78%) discriminated American black bear from brown (grizzly) bear scats even when the bears were fed the same diet. These two studies are in accordance with our study, which shows that dogs are able to discriminate the two beaver species with high accuracy (90%).

Oldenburg, *et al*.^37^ recently showed that it was possible to train one dog on only two variations of spraints from captive otters (*Lutra lutra*) so that it could recognize and respond to wild scat samples, even if the diets of the otters differ. This indicates that the dog can rapidly generalize variations of otter spraint scent of which it has not been trained on, i.e. from very few samples to many and from captive diet to wild. Our dogs also showed this trait. During training, we used beaver castoreum samples from which some of the more volatile compounds were no longer present as they had been stored in the freezer for 15 years (Tinnesand and Rosell unpublished). Yet in the second experiment, the dogs were able to recognize scent marks from wild beavers.

This study demonstrates that the composition of compounds present in castoreum differs between the two beaver species. These differences are probably due to genetic factors through geographical isolation. The two species inhabit similar vegetation types^38^ and probably forage on many of the same plants^39,40^. Therefore, the most obvious explanation to account for the difference in chemical composition would be the differences in their genetics^41^ or bacterial flora in their castor sacs^42^. The two beaver species occur in close proximity in Finland (K. Kauhala pers. Comm.)^14^, but it was not possible to collect *Cf* scent samples from areas where they occur in close proximity or overlap in Finland. Therefore, we cannot rule out that diet may be partly responsible for the observed difference since castoreum is a mixture of secondary metabolites most likely originating from the beavers diet^43^. As the scent samples in our study originated from two beaver species which differed genetically and geographically, the next step should therefore be to test if dogs respond differently to castoreum scents collected where the two beaver species share the same habitat.

Currently the most accurate methods for species identification requires anal gland secretion or DNA (-see introduction), which are both costly and time consuming. These methods require extensive laboratory time for analyses or field work for trapping (e.g. by use of Hancock live-traps), which makes them inappropriate when immediate management action is needed. Our results show that trained scent detecting dogs can be an efficient and feasible solution in practical management. Our tests were all carried out in a laboratory environment. The next step should therefore be to rigorously test if dogs discern between beaver species in the field under a range of weather conditions and habitat types. Whilst training dogs is time consuming and expensive, once the dogs are imprinted on a scent, their detection ability are immediate in the field. Also, using trained dogs can be cost-effective, if carried out in co-operation with existing public agencies such as the police who already have dogs trained for detection operations (see e.g. Orkin *et al*.^29^).

Dogs have often been used to find and identify scats from animals in North and South America^27,44–46^, Asia^29^ and Australia^47,48^. However, in Europe, as far as we know, only two studies have been carried out^33,37^. We also point out that dogs do not necessarily need to be brought into the field but samples, i.e. scent marks or feces, can be brought to the laboratory^49,50^.

Our results, taken with the broader literature, confirm that dogs can be used as an “animal biosensor” in a laboratory setting to discriminate between scent samples from similar species (same genus) of many different taxa. Our study results show that dogs can be used as a promising tool to promote conservation of the native beaver species and eradication of the invasive one. We therefore conclude that, dogs may be an efficient non-invasive tool to help conservationist to manage invasive species in Europe, and advocate for European wildlife agencies to invest in this new tool.

## Material and Methods

### Dogs

We used seven privately owned dogs as “animal biosensors”^51^ (Table 1). All had a basic level of obedience but were naïve to scent detection work. None of the females were in heat or pregnant during the experiments (Table 1). The dogs were randomly assigned to a species, i.e. *Cc* or *Cf*, and four dogs were trained to recognize castoreum from *Cf* and three dogs to recognize castoreum from *Cc* (Table 1).

**Table 1:** The dogs trained for the experiments, the beaver species they were imprinted on (castoreum), as well as their sex, breed, age at final experiment and the handler (all inexperienced).

Dog	Species imprinted	Sex	Breed	Age	Handler
Chilli	Eurasian	Female	Border Collie	7	A
Danny	Eurasian	Male	Samoyed	5	B
Tapas	Eurasian	Male	Border Collie	7	A
Triana	Eurasian	Female	Papillion	3	B
Bailey	North American	Male	Nova Scotia Duck Tolling Retriever	4	C
Carmeita	North American	Female	Papillion	3	B
Shib	North American	Female	Border Collie	10	C

### Scent donors

*Cf* castoreum samples were collected from nine adult males (mean weight ± SD (kg) = 20 ± 2.09 kg) and seven adult females (20 ± 2.6 kg) that were shot in twelve different territories in Bø, Nome and Sauherad municipalities, Norway, during the normal hunting season (1^st^ October - 30^th^ April 1999). The *Cc* castoreum samples were collected from nine males (19 ± 2.9 kg) and seven adult females (20 ± 2.3 kg) that were shot in 8 different territories during the normal hunting season (20 August – 30^th^ April) in the South Savo Game Management District in central Finland during 1999 and 2000 (S. Härkönen pers. comm.). The castoreum samples were identified, both sex and species, by the color and the viscosity of their anal gland secretion^21^.

All shot beavers were frozen (−20 °C) immediately after death until dissection of the animals could be performed. The extraction of castoreum was performed by making a lateral incision through the outer layer of the castor sacs, thereby revealing the castoreum, which was then removed from the pocket lumen^52^.

Experiment one used 20 castoreum samples from dead beavers (10 from each species). Experiment two used 18 beaver castoreum scent marks (nine from each species) collected from nine different territories in Bø, Norway, and from eight different territories in Evo, Finland. The scent samples from Finland, were collected from an area where all the sampled individuals have been determined to be *Cc* based on skull morphology and DNA analyses from hunted individuals. Castoreum samples from dead beavers and castoreum scent marks used in testing trials during experiments had not been used in training.

Disposable gloves were worn to avoid scent contamination and all castoreum samples and scent marks were stored in glass vials with teflon lids (57 × 27.5 mm, Qorpak®, Pennsylvania, USA) and stored in a freezer at −20 °C until used in the dog scent detection training or experiments.

### Ethical note

All methods were performed in accordance with the relevant guidelines and regulations of our university. Further approvals from other ethics commitees or ethics boards were not required. No animals did experience anesthesia, euthanasia or any kind of sacrifice as a part of this study.

### Dog training

**Figure 1:**
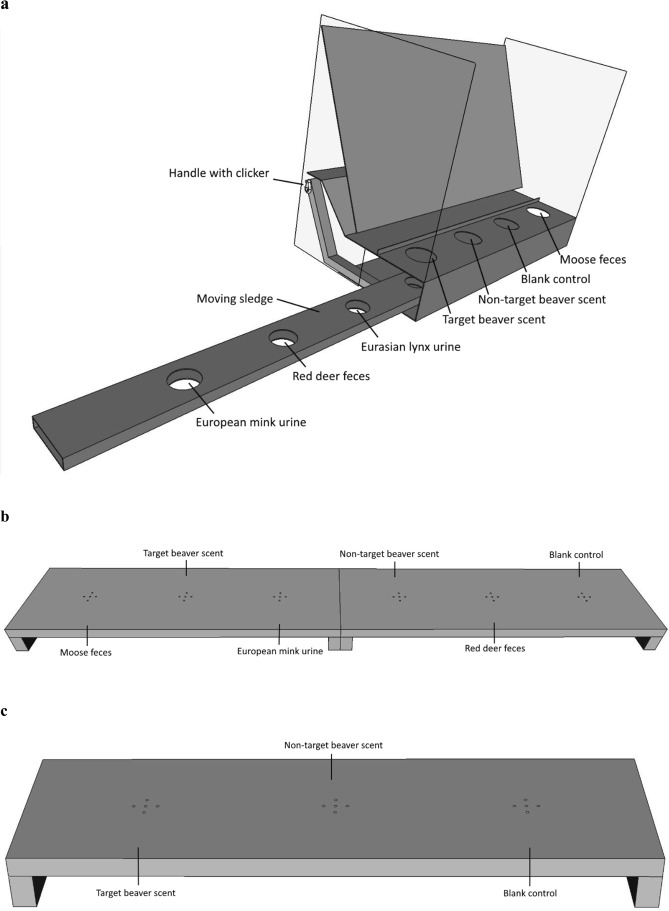
(**a–c**) The platforms used in training and experiments: (**a**) the table platform with the sledge containing seven cups was used in training phases one and two so that four cups are presented at one time, (**b**) the full floor platform used in training phase three and experiment one and (**c**) the floor platform used in training phase four and experiment two. Note that all samples were randomly placed in the platforms during training and experiments.

All training was carried out in a laboratory at University College of Southeast Norway, Bø in Telemark, Norway. Training started in October 2013 and was carried out periodically until April 2015. In total, the dogs participated in 60 training sessions each lasting about two hours with each dog training for approximately 15 minutes. The training was split into four phases, (1) adaptation to the laboratory and training methods on the table platform, (2) imprinting on a beaver species (*Cf* or *Cc*) and discrimination training, (3) adaption from the table platform to the floor platform, and 4) absence of target scent training. Our training was based on positive reinforcement^53^, where the dogs were taught to associate the sound of a clicker with a food reward^54^, and the handlers also petted and vocally rewarded the dogs^55^. In general, we followed the recommendations for dog training and experimental design from Johnen, *et al*.^56^. All random selections within training were carried out with the random number generator in Microsoft Excel®. We used a table platform for initial training and a floor platform for both training and experimental procedures. The table platform is adapted from a standard method developed by Hundcampus training center, Hällefors, Sweden (Fig. 1a, see also Fischer-Tenhagen, *et al*.^57^ for table platform details). The floor platform (detection board) is commonly used in dog scent detection training and experiments (Fig. 1b)^58^. All equipment was cleaned with vinegar and water between training trials.

#### Phase one - adaptation

Phase one began with introducing the dogs to the table platform and teaching them to associate the sound of a clicker with a reward. A food treat (AB Dogman, Klassiskt. Åstorp, Sweden) was used as a target scent to train the dogs to sniff each of the plastic cups in the setup. The table platform presented four samples at a time to the dog (Fig. 1a). A plastic cup containing a cotton pad and a food treat was placed opposite the handle of the sledge (dogs could therefore not see the handle) and clicker, and empty plastic cups were placed in the other six locations. A perforated metal plate between the platforms cover panel and the samples was used, to avoid the samples being visible to the dogs. The dogs were trained to investigate the scent by being rewarded immediately when sniffing the plastic cup containing the food treat. The sledge containing the plastic cups was then moved from side to side and the dog was told to “search” so that the dog could find the food treat in different locations. Once the dog was competent at locating the food treat, blank cotton pads were placed in the other plastic cups to ensure the dog was targeting the food treat scent^57,58^.

Phase one then progressed to teaching a dog to indicate the location of the food treat by standing still and pointing at the scent with its nose. Initially the dogs were rewarded immediately, but the moment of reward was continuously prolonged, until the dogs lay down with their nose over the food treat. Once the dogs had learned to correctly indicate the food treat, distraction scents were placed in the empty plastic cups. In each cup, there was either 0.4 g of feces from moose (*Alces alces*), red deer (*Cervus elaphus*), or roe deer (*Capreolus capreolus*) that had been collected from nine different individuals of each species, or a cotton pad containing four drops of urine from either European mink, red fox, or Eurasian lynx (*Lynx lynx*) (all urine was purchased from www.pelsjeger.no). Thus, the dogs learned to investigate all four plastic cups presented at a time, in order to identify the target scent among the distraction scent. This pre-training was considered accomplished following 20 consecutive correct indications with the dogs indicating the food treat among the distraction scents by lying and pointing with its nose for at least three seconds^58^. All dogs achieved this goal in four sessions of 15 minutes over a two-week period.

#### Phase two – imprinting and discrimination

In phase two, the dogs were first trained to imprint on castoreum from dead *Cf* or *Cc*, and then tested via trials as to whether they could discriminate between them.

### Training on target scent

For training, 0.4 g of the target castoreum scent (*Cf* or *Cc*) was placed in a new glass vial and placed in the plastic cup opposite the handle of the sledge on the table platform. This was first repeated 20 times with empty glass vials in the other six cups. Then, a glass vial with 0.4 g of castoreum of the non-target species was placed in one of the empty plastic cups, an empty glass vial was placed in another cup and distractions, similar to those in phase one, were placed in the remaining four cups. As in phase one, phase two progressed to include distraction scents of feces and urine from other species (see above). All equipment was cleaned with water and vinegar between training trials.

### Discrimination training trials

**Figure 2:**
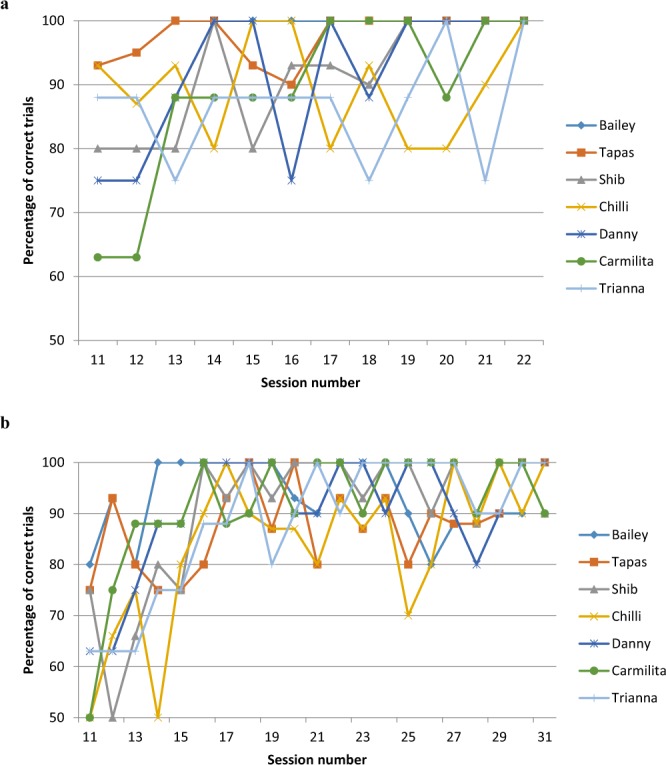
(**a,b**) Percentage curves of the correct trials for each dog during 12 training sessions with the table platform. The percentage curves begin from training session 11 once the dogs were imprinted on their target beaver scent (**a**) and during 16 training sessions with the floor platform from session 11 of training, once the dogs had adapted to the new platform (**b**). All these sessions were single blind test.

One positive and six negative samples were included in each trial. At this stage the handlers were blind to the location of the target scent and an experimenter, not blind to the location of the target scent (single blind), confirmed the dog’s accuracy^59^. In each trial different samples were used to ensure dogs did not imprint on individual beavers^60^. Each session was composed of 8–16 trials to ensure that the dog could discriminate between the castoreum of the dead beaver, and dogs were always rewarded for indicating the correct glass vial. During phase two, training outcome was recorded during 12 sessions using the table platform once the dogs were imprinted on their target scent (Fig. 2a).

#### Phase three – adaptation to floor platform

In phase three, we used a floor platform as a training device, in which six scents were lined up, and the dogs needed to pass each scent to detect the target scent, a method regularly used in scent detection training and testing (Fig. 1b)^61–63^. Apart from the change in training device, the method was the same as phase two. During phase three, training outcome was recorded during 16 sessions once the dogs had adapted to the floor platform (Fig. 2b). In total, each dog participated in 32 training sessions before experiment one.

#### Phase four – absence of target scent training trials

In phase four, in which four dogs participated, we removed half of the floor platform so that only three scents were presented to the dogs, and we introduced blank and non-target scent trials (i.e. no target scents, Fig. 1c). If the target castoreum was not located in the platform, the dogs were trained to return to the handler and sit to indicate that no target castoreum was present. The dogs work in a similar manner to the table platform, but to avoid any influence from the handler, a screen was used, which the handler stands behind, so that the dog works alone to avoid the “Clever Hans effect”^64^. The four dogs participated in 27 training sessions between experiment one (i.e. discrimination between the two beaver species using castoreum samples from dead beavers) and experiment two (i.e. discrimination between the two beaver species via castoreum scent marks, and ignoration of the blank and non-target scent trials).

### Experiments evaluating the accuracy of scent discrimination

To evaluate the accuracy of the dogs to discriminate between *Cf* and *Cc* castoreum, two different double-blind experiments were carried out using the floor platform over three separate sessions on June 25–26, 2014 (experiment one, four dogs), April 15–16, 2015 (experiment one, three dogs) and May 8–10, 2015 (experiment two, four dogs). All samples for both experiments were used once for each dog and their placements in the setup were randomized using the random number generator in Microsoft Excel®.

All equipment was cleaned with vinegar and water between testing trials and new plastic cups were used. The handler stepped behind a screen, and was blind to the position of the castoreum scent marks^56^. The experimenter was not present in the room, and all testing trials were documented using three tripod-mounted video cameras (Sony DCR-SR35E), that was set to record continuously, placed on either side, and in front of the platform, to ensure all behaviors were visible. An independent referee watched the videos of the experiments to determine the number of correct testing trials and to ensure that the experiment was double-blind^56^.

In experiment one, using the full floor platform, a glass vial containing 0.4 g of castoreum from a dead *Cc* was placed in a plastic cup and a glass vial containing 0.4 g from a dead *Cf* was placed into a separate plastic cup. In addition to the target castoreum (*Cf* or *Cc*) and distraction castoreum (*Cf* or *Cc*), the dogs were presented with a glass vial containing 0.4 g moose feces in a plastic cup, a glass vial containing 0.4 g red deer feces in a plastic cup, a blank glass vial in a plastic cup, and an empty plastic cup. Feces samples used in experiments had not been previously used for training and were from 10 individuals of each species to prevent familiarity of scent. A total of 10 testing trials were run per dog.

For experiment two, only half of the floor platform was used, as with phase four of the training, so that only three scents were presented in each testing trial and we used castoreum scent-marks from beavers. Each testing trial had one of three options, (1) the target castoreum, the non-target castoreum and a blank, (2) the non-target castoreum and two blanks, (3) three blanks. A total of 27 testing trials were run per dog, nine per each trial option that were presented in a randomly selected order using the random number generator in Microsoft Excel®.

The four possible responses of the dogs in the experiments were recorded as:

1. True positive response (TP), i.e. the dog indicated on the target beaver species.
2. False negative response (FN), i.e. the dog did not indicate on the target species castoreum.
3. True negative response (TN), i.e. the dog did not indicate on the non-target beaver species.
4. False positive response (FP), i.e. the dog indicated on a blank or non-target species castoreum.

From these responses three parameters were calculated to determine whether the dogs can discriminate between the castoreum of *Cc* and *Cf*: sensitivity, specificity and accuracy^51,65,66^.

*Calculation of sensitivity: TP/(TP* + *FN)*,

*Calculation of specificity: TN/(TN* + *FP)* and

*Calculation of accuracy: (TP* + *TN)/(TP* + *FP* + *TN* + *FN)*.

## Data Availability

Appropriate data will be uploaded on Dyrad respository upon acceptance.
